# The predictive value of pressure recording analytical method for the duration of mechanical ventilation in children undergoing cardiac surgery with an XGBoost-based machine learning model

**DOI:** 10.3389/fcvm.2022.1036340

**Published:** 2022-10-28

**Authors:** Mingwei Li, Shuangxing Wang, Hui Zhang, Hongtao Zhang, Yongjie Wu, Bing Meng

**Affiliations:** Department of Cardiac Surgery, Children's Hospital of Capital Institute of Pediatrics, Beijing, China

**Keywords:** CHD, congenital heart disease, cardiac surgery, pressure recording analytical method, mechanical ventilation, machine learning model

## Abstract

**Objective:**

Prolonged mechanical ventilation in children undergoing cardiac surgery is related to the decrease in cardiac output. The pressure recording analytical method (PRAM) is a minimally invasive system for continuous hemodynamic monitoring. To evaluate the postoperative prognosis, our study explored the predictive value of hemodynamic management for the duration of mechanical ventilation (DMV).

**Methods:**

This retrospective study included 60 infants who underwent cardiac surgery. Cardiac index (CI), the maximal slope of systolic upstroke (dp/dt_max_), and cardiac cycle efficiency (CCE) derived from PRAM were documented in each patient 0, 4, 8, and 12 h (T0, T1, T2, T3, and T4, respectively) after their admission to the intensive care unit (ICU). A linear mixed model was used to deal with the hemodynamic data. Correlation analysis, receiver operating characteristic (ROC), and a XGBoost machine learning model were used to find the key factors for prediction.

**Results:**

Linear mixed model revealed time and group effect in CI and dp/dt_max_. Prolonged DMV also have negative correlations with age, weight, CI at and dp/dt_max_ at T2. dp/dt_max_ outweighing CI was the strongest predictor (AUC of ROC: 0.978 vs. 0.811, *p* < 0.01). The machine learning model suggested that dp/dt_max_ at T2 ≤ 1.049 or < 1.049 in combination with CI at T0 ≤ 2.0 or >2.0 can predict whether prolonged DMV (AUC of ROC = 0.856).

**Conclusion:**

Cardiac dysfunction is associated with a prolonged DMV with hemodynamic evidence. CI measured by PRAM immediately after ICU admission and dp/dt_max_ 8h later are two key factors in predicting prolonged DMV.

## Introduction

Myocardial dysfunction is a critical challenge for hemodynamic management after cardiac surgery. For infants with congenital heart disease, the continuous decline in cardiac performance may remain long after cardiopulmonary bypass (CPB) (1, 2). Cardiac index (CI) decreased around 30% postoperatively in neonates, reaching the lowest point 9–12 h after surgery (1). Adults show decreasing CI over the first 4–8 h in the ICU but recover to the baseline within 24 h (3). Hemodynamic instability and myocardial dysfunction in infants often cause serious complications, which incur higher mortality, prolong the cardiopulmonary support, and even lead to weaning failure (4, 5).

As cardiac surgeons have widely admitted the duration of mechanical ventilation as an index for patients' prognosis after surgery, many studies have tried to reveal the risk factors associated with delayed extubation. However, most of these studies focused on the direct effects of the respiratory system on mechanical ventilation and failed to consider about the radical hemodynamic changes and heart–lung interactions as a whole (6–8). Though a few studies suggested that weaning failure is associated with increased left ventricular end-diastolic pressure (LVEDP) and left ventricular dysfunction (9, 10), clinical explorations based on the theory of the heart–lung interactions in the perioperative period still lack data evidence and methods on evaluating the appropriate timing for weaning remain limited (11, 12). With the benefits of early extubation wellestablished, fast-track anesthesia is now widely used in cardiac 40 surgery in adult, but less well described in children (13–15). Therefore, it is of great necessity for feasible evaluation on the course of mechanical ventilation.

Our research aimed to reveal that hemodynamic parameters representing cardiac function at the early postoperative time can predict the duration of mechanical ventilation, which provides new insights into postoperative management.

## Patients and methods

### Patients

This retrospective study included 60 infants who underwent heart defect repaired surgeries in our hospital between January 2017 and March 2021. Patients who expired during hospitalization were excluded. All the open heart surgeries were performed through standard procedures of CPB. Mechanical ventilation was initiated immediately when the patient arrived at the ICU after surgery. Extubation was performed when the patients met the standard criteria: (1) hemodynamic stability with reasonable urination and warm peripheral extremities; (2) PaO_2_ ≥75 mmHg and PaCO_2_ ≤ 50 mmHg with adequate spontaneous respiration under FiO_2_ ≤ 40% and the end of expiratory pressure ≤ 5 cm H_2_O; (3) awake and able to respond to commands without new neurological symptoms; (4) no active bleeding with a reasonable change in hemoglobin and no requirement for volume replacement; and (5) no reasonable fear of reintubation. The duration of mechanical ventilation (DMV) longer than 24 h was considered with delayed extubation, and patients were divided into two groups according to this.

### Hemodynamic monitoring

Postoperative management consists of continuous intravenous infusion of sufentanil, dexmedetomidine, or midazolam. Inotropic and vasoactive drugs include dopamine, milrinone, epinephrine, norepinephrine, or levosimendan to maintain arterial blood pressure. Anti-infective treatment is routinely performed with the intravenous antibiotic. Since the Fick (1870) principle was developed (16), many technological methods have been invented to measure the cardiac output and other hemodynamic parameters. MostCare (Vygon Vytech, Padova, Italy) uses the pressure recording analytical method (PRAM) for direct monitoring based on pulse spectrum analysis methods in the same way as PICCO_2_. It is a minimally invasive real-time method recorded at a high sampling rate (1,000 pressure/time points) without the need of calibration (17, 18). Most care has shown a good level of agreement with the Fick method measurements and is widely studied in animals and adults but rarely in infants after cardiac surgery. Hemodynamic parameters collected in this study included cardiac index (CI), the maximal slope of systolic upstroke (dp/dt_max_), and cardiac cycle efficiency (CCE). Data were collected and recorded by the device 0, 4, 8, and 12 h after radial artery cannulation was established (T0, T1, T2, and T3, respectively).

### Analysis method

The Shapiro–Wilk method was used to test whether the data followed the normal distribution that were normally distributed and median values with interquartile range (IQR, 25th−75th percentile) for variables that were not normally distributed, and as the frequency with percentage (%) for categorical variables. Hemodynamic data recorded by MostCare are presented as mean ± SD. In univariate analysis, differences between groups were evaluated using the Wilcoxon rank-sum test or *t*-test for continuous variables according to distribution. Chi-square test and Fisher's exact test are used for categorical variables. Linear mixed models were used to deal with the repeated measurement of hemodynamic data. For each CI, CCE, and dp/dt_max_, we tested for interactions between groups (DMV ≤ 24h or >24h) and time (T0, T1, T2, and T3). The model equation is shown as follows:


$$\begin{array}{l} {\text{Y}}_{\text{i},\text{j}\;}={\text{β}}_{\text{0}}{\text{+β}}_{\text{1}}{\text{ (Time) +β}}_{\text{2}}\;\left(\text{Group}\right)\;+{\text{β}}_{\text{3}}\;\left(\text{Group}\times \text{Time}\right)\;\\ \;+{\text{b}}_{0}\;\left(\text{ID}\right)\;+{\text{b}}_{\text{1}} \end{array}$$

where Y_i, j_ is the hemodynamic variables (CI, CCE, or dp/dt_max_) for patients i at Time j (*i* = 1, 2,…60. *j* = 0, 1, 2, 3); Time^*^Group is the interaction between the group term and the time term. ID is the patients i. β_0_, β_1_, β_2_, and β_3_ are the fixed effect coefficients. b_0_ and b1 are the random effect coefficients. The Pearson correlation coefficient was calculated to assess the relationship between the cardiac functions and DMV. ROC curves are used to assess the diagnostic performance. *p*-value < 0.05 was considered statistically significant. Statistical analysis and data processing were performed with *R* language (version 4.2.0).

### Machine learning model

Yan et al. (20) have designed an XGBoost machine learning-based model that can predict the 92 mortality rates of patients with more than 90% accuracy for COVID-19 prognostic prediction. The treeheatr *R* package creates interpretable decision tree visualizations with the data represented as a heat map at the tree's leaf nodes (21). XGBoost algorithms are based on recursive decision tree building from past residuals and can identify those trees that contribute the most to the decision of the predictive model. The leaf nodes are labeled based on their majority votes and colored to correlate with the true outcome in the decision tree. The models were evaluated by assessing the classification accuracy (ratio of true predictions overall predictions), the precision, sensitivity/recall, and defined scores. The importance of individual feature in XGBoost is determined by its accumulated use in each decision step in trees, computing the relative importance of each feature. Hence, it can estimate features that are the most discriminative of model outcomes. Using this machine learning model, we construct a clinically operable decision model.

## Results

### Patients' characteristics

Demographic characteristics are shown in Table 1. A total of 60 infants were included in the study, among which 35 (58%) children were extubated within 24 h (DMV ≤ 24h group), while 25 (42%) were over 24 h (DMV>24h group). According to the pathophysiology, congenital heart disease (CHD) is divided into left-right shunt CHD and right-left shunt ones. In our study, the former included atrial septal defect (ASD), ventricular septal defect (VSD), patent ductus arteriosus (PDA), and simple valvular disease, while the latter included tetralogy of Fallot (TOF), double outlet right ventricle (DORV), and Complete endocardium pad defect (CEPD). Infants with a prolonged DMV were characterized by significantly younger ages, lower heights and weights, longer CPB time and aortic cross clamp (ACC) time, as well as longer ICU and postoperative hospital stays (*p*-value < 0.05) (Table 1).

**Table 1:** Baseline characteristics.

**Characteristics**	**DMV ≤ 24h**	**DMV > 24h**	***p*-value**
	**(*n* = 35)**	**(*n* = 25)**	
Age (month)	4.77 (3.50, 6.86)	2.00 (1.57, 4.53)	**< 0.001**
Sex			0.493
Female	11 (31%)	10 (40%)	
Male	24 (69%)	15 (60%)	**0.002**
Height (cm)	64.51 ± 5.75	58.76 ± 7.45	
Weight (kg)	6.2 (5.50, 7.40)	5.00 (4.20, 6.20)	**0.001**
CHD*			1.000
Left–right	31 (89%)	22 (88%)	
Right–left	4 (11%)	3 (12%)	0.660
NYHA			
≤ II	19 (54%)	15 (60%)	
>II	16 (46%)	10 (40%)	0.357
Preoperative respiratory disease**			
Yes	14 (40%)	13 (52%)	
No	21(60%)	12(48%)	**0.004**
CPB time (min)	76 (66, 92)	104 (86, 136)	**0.016**
ACC time (min)	45 (36, 56)	55 (50, 79)	**< 0.001**
Ventilation time (h)	12 (8,20)	49 (45,72)	0.199
Adverse events***			
Yes	18 (51%)	17(68%)	
No	17 (49%)	8(32%)	**< 0.001**
ICU stay (days)	2 (1,3)	5 (4,7)	**< 0.001**
Postoperative hospital stay (days)	10 (9,12)	14 (12,18)	

Data are presented as mean(IQR)/*n* (%)/mean±SD. The bold values represent the *p*-value < 0.05.

DMV, duration of mechanical ventilation; CPB, cardiopulmonary bypass; ACC, aortic cross clamp; NYAH, NYAH class.

*CHD, congenital heart disease includes left-right shunt congenital heart disease and right-left shunt congenital heart disease.

**Preoperative respiratory disease includes respiratory tract infection, pneumonia, and tracheal chondromalacia.

***Adverse events, whether the patient is subjected to one of the following postoperative complications during the hospitalization: Low cardiac output syndrome, pleural effusion, ascites, arrhythmia, infection, or pneumonia.

### Hemodynamic data of the two DMV groups

**Figure 1:**
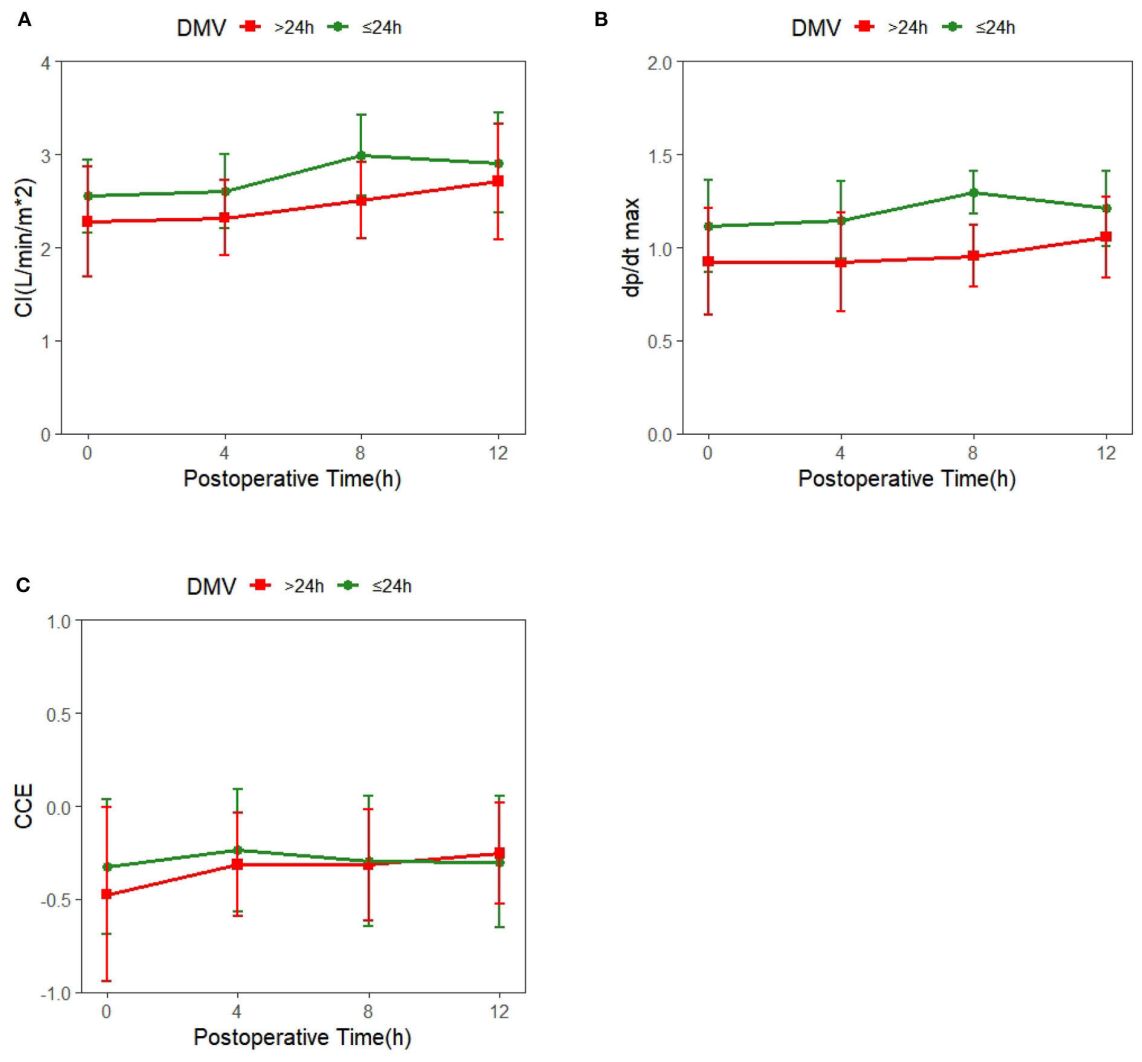
Trends of systemic hemodynamic value over time in patients with different duration of mechanical ventilation (DMV); the red line represents DMV >24h, and the green line represents DMV ≤ 24h. **(A)** Time-dependent changes in cardiac index(CI). **(B)** Time-dependent changes in cardiac cycle efficiency (CCE). **(C)** Time-dependent changes in the maximal slope of the systolic upstroke (dp/dt_max_).

Mean ± SD of hemodynamic monitoring in the ICU at different time points for each DMV group was recorded (Supplementary Table 1), and the changes in cardiac function over time were shown (Figure 1). The results from the linear mixed model revealed significant main effects of time and group in CI and dp/dt_max_, but not CCE (Table 2). DMV ≤ 24h group showed significant increases in CI and dp/dt_max_ from T0 to T2 (CI, β_1_ = 0.44, SE = 0.09, *p* < 0.001; dp/dt_max_, β_1_ = 0.182, SE = 0.049, *p* < 0.001) and from T0 to T3 (CI, β_1_ = 0.35, SE = 0.09, *p* < 0.001; dp/dt_max_, β_1_ = 0.096, SE = 0.049, *p* < 0.05). T0 observed a significant difference between the two groups with decreased CI and dp/dt_max_ in patients with a prolonged DMV (CI, β_2_ = −0.27, SE = 0.12, *p* < 0.05; dp/dt_max_, β_2_ = −0.182, SE = 0.058, *p* < 0.01). Besides, there was a significant group-time interaction in dp/dt_max_ from T0 to T2 (β_3_ = −0.152, SE = 0.075, *p* < 0.05) but not CI or CCE.

**Table 2:** Linear mixed effects of hemodynamic variables.

	**CI**	**CCE**	**dp/dt_max_**
**Fixed effects**			
_T0_	2.55(0.08)**	−0.327(0.057)	1.113(0.035)**
_T0−*T*1_	0.04(0.09)	0.090(0.065)	0.034(0.048)
_T0−*T*2_	0.44(0.09)***	0.031(0.065)	0.182(0.049)***
_T0−*T*3_	0.35(0.09)***	0.028(0.065)	0.096(0.049)*
_Group × *T*0_	–0.27(0.12)	–0.148(0.091)	–0.182(0.058)
_Group × *T*0−*T*1_	–0.004(0.14)	0.070(0.100)	–0.038(0.075)
_Group × *T*0−*T*2_	–0.21(0.14)	0.128(0.100)	–0.152(0.075)*
Group × T0–T3	0.08(0.14)	0.192(0.100)	0.036(0.075)
**Random effect**			
Individual	0.08(0.28)	0.042(0.20)	0.002(0.046)
Group × Individual	0.29(0.54)	0.064(0.27)	0.022(0.148)
Corr.	–0.96	–0.52	–1.00
Log Likelihood	–154.1	–75.41	15.37
AIC	332.21	174.81	–6.73
BIC	373.98	216.58	35.03

Values indicate estimated effect β and corresponding standard error.

CI, cardiac index; CCE, cardiac cycle efficiency; dp/dt_max_ the maximal slope of the systolic upstroke.

*p < 0.05.

**p < 0.01.

***p < 0.001.

### Correlation analysis

Age, height, weight, CPB, ACC, which showed a significant difference between the two groups by univariate analysis (Table 1), and CI and dp/dt_max_ at T0, T2, and T3, which showed significant effects of time or group in the linear mixed models (Table 2), were entered for correlation analysis. Figure 3 shows a prolonged DMV has significant and negative correlation with age (*r* = −0.48, *p* < 0.01), weight (*r* = −0.42, *P* < 0.05), CI at T2 (*r* = −0.53, *p* < 0.001), and dp/dt_max_ at T2 (*r* = −0.82, *P* < 0.001). There was no significant correlation in CPB or ACC. dp/dt_max_ at T2 has a strong correlation, whereas age, weight, and CI at T2 have a moderate correlation.

### Predictive values

#### ROC curves

As shown in Figure 3, dp/dt_max_ outweighing CI at T2 was the strongest predictor of a prolonged DMV (*p* < 0.01). dp/dt_max_ at T2 < 1.052 (sensitivity = 1.000, specificity = 0.840), CI at T2 < 2.670 (sensitivity = 0.800 specificity = 0.800), and CI at T0 < 2.215 (sensitivity = 0.857 specificity = 0.560) could predict prolonged DMV.

#### XGBoost machine learning-based model

Age, height, weight, CPB, ACC, CI, and dp/dt_max_ at T0, T2, and T3 were also entered into the XGBoost machine learning-based model, which produced a decision tree-heat map. As Figure 4 shows, the model suggested that patients with dp/dt_max_ ≤ 1.049 at T2 were in DMV>24h group, and those whose dp/dt_max_ > 1.049 at T2 was in DMV ≤ 24h. On the split of CI at T0, although individuals of both branches are all predicted to DMV ≤ 24h by majority voting, the leaf nodes have different purity, indicating different confidence levels the model has in classifying samples in the two nodes. Therefore, patients with CI ≤ 2 at T0 cannot easily exclude the possibility of being prolonged DMV. The whole model had excellent accuracy and predictive value (accuracy = 0.933, balance accuracy = 0.920, Kappa = 0.860, AUC of ROC = 0.856, AUC of PR = 0.907).

## Discussion

Andre' and DelRossi reported that PRAM has been proved to correlate well with “gold-standard” thermodilution (3) in assessing cardiac output. Other methods included Fick, Doppler echocardiography, BNP, and lactate levels (19, 22). Our study initiatively found correlations between the cardiac function characterized by hemodynamic parameters and mechanical ventilation. Prolonged DMV, mainly caused by cardiac dysfunction, is a significant sign of worse prognosis, and successful early extubation is a goal to promote recovery after cardiac surgery with both medical and economic benefits (13, 14). Furthermore, we proved good predictive value of these parameters and provided a visualized decision-making map with the application of machine learning model.

### Hemodynamic management with PRAM in the postoperative process

The linear mixed model showed time effects of hemodynamic parameters, indicating that cardiac function presented a significant increase over time in the first 4–8h after the surgery (Figure 1 and Table 2), which contradicted the classic conclusions from Wernovsky et al. (1) and Gil-Anton et al. (4) measured CI of children with CHD 24 h after surgery by femoral arterial thermodilution and found no obvious changes between CI over time. Another exploration of trends of the postoperative hemodynamic based 161 on PRAM found an increase in CCE and dp/dt_max_ in 48 h postoperatively (23). The different trends of postoperative cardiac function between the early study and later ones perhaps resulted from the developed CPB techniques, such as modified ultrafiltration and dexmedetomidine sedative (24–26), which may prevent CPB-related inflammatory responses and lead to better hemodynamic outcomes. Besides, the use of vasoactive agents has proven to be another key factor contributing to improving postoperative cardiac function (27–29).

### Cardiac function for predicting DMV

**Figure 2:**
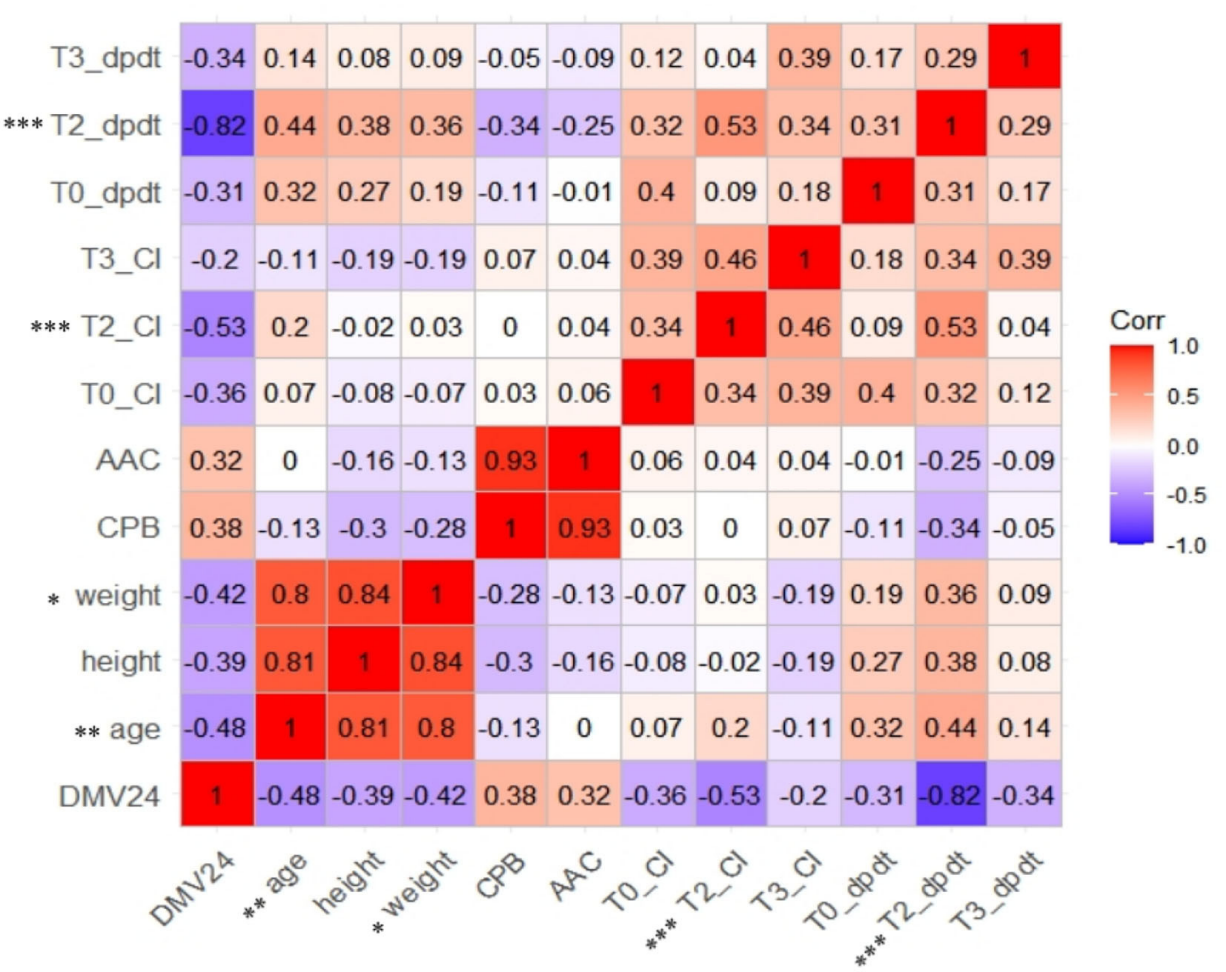
Correlation analysis of heat map for relationship between selected the characteristics, hemodynamic parameters, and prolonged DMV. CI, cardiac index. Dp/dt max, the maximal slope of systolic upstroke. DMV24, binary-classified variable, duration of mechanical ventilation leq24h or >24h.

**Figure 3:**
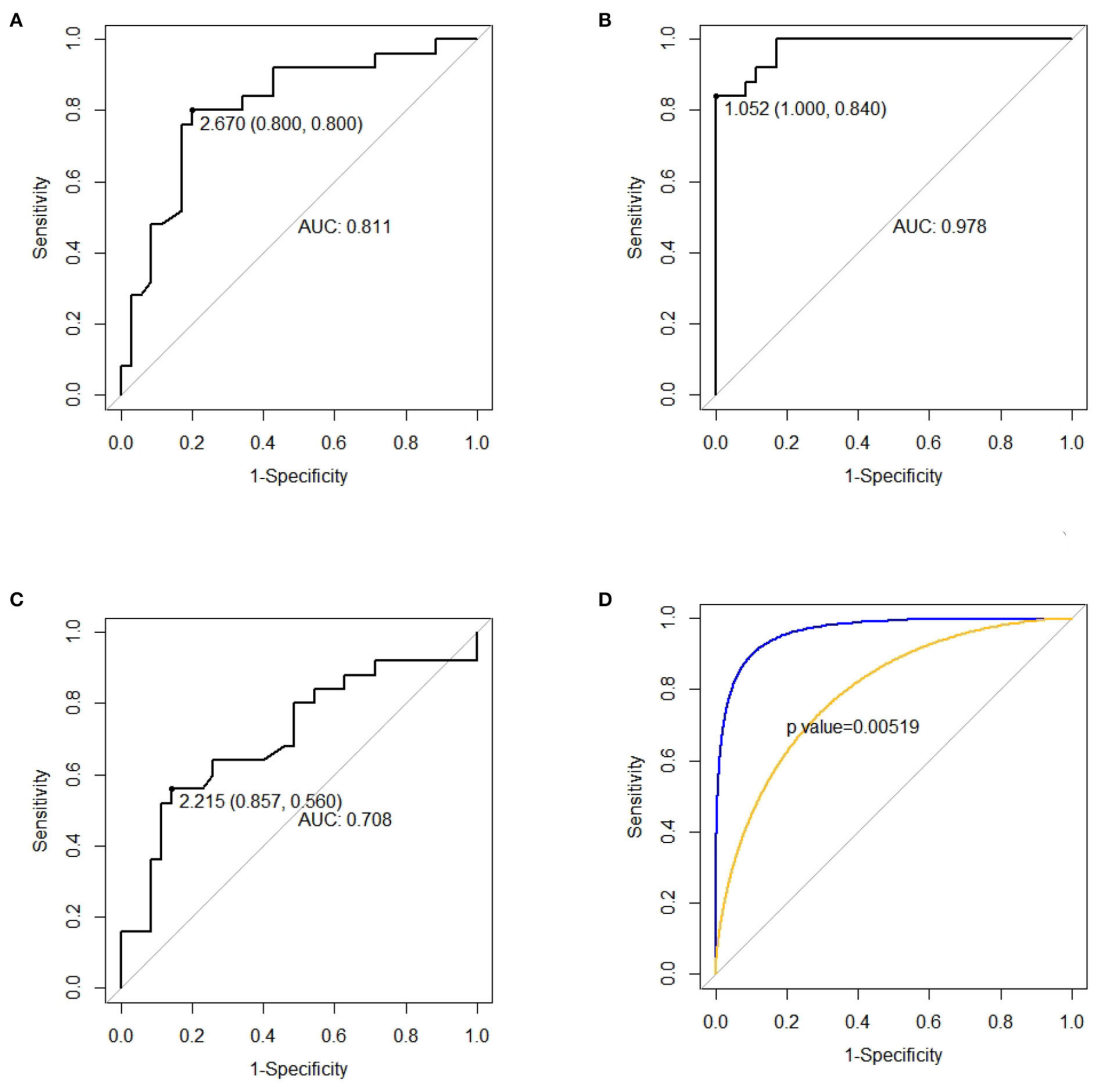
ROC curves for predicting prolonged duration of mechanical ventilation (DMV). **(A)** CI, cardiac index, measured at T2 for predicting prolonged DMV. **(B)** dp/dt_max_, the maximal slope of the systolic upstroke, measured at T2 for predicting prolonged DMV. **(C)** CI, cardiac index, measured at T0 for predicting prolonged DMV. **(D)** Comparison between the AUC of ROC of CI and dp/dt_max_ at T2.

**Figure 4:**
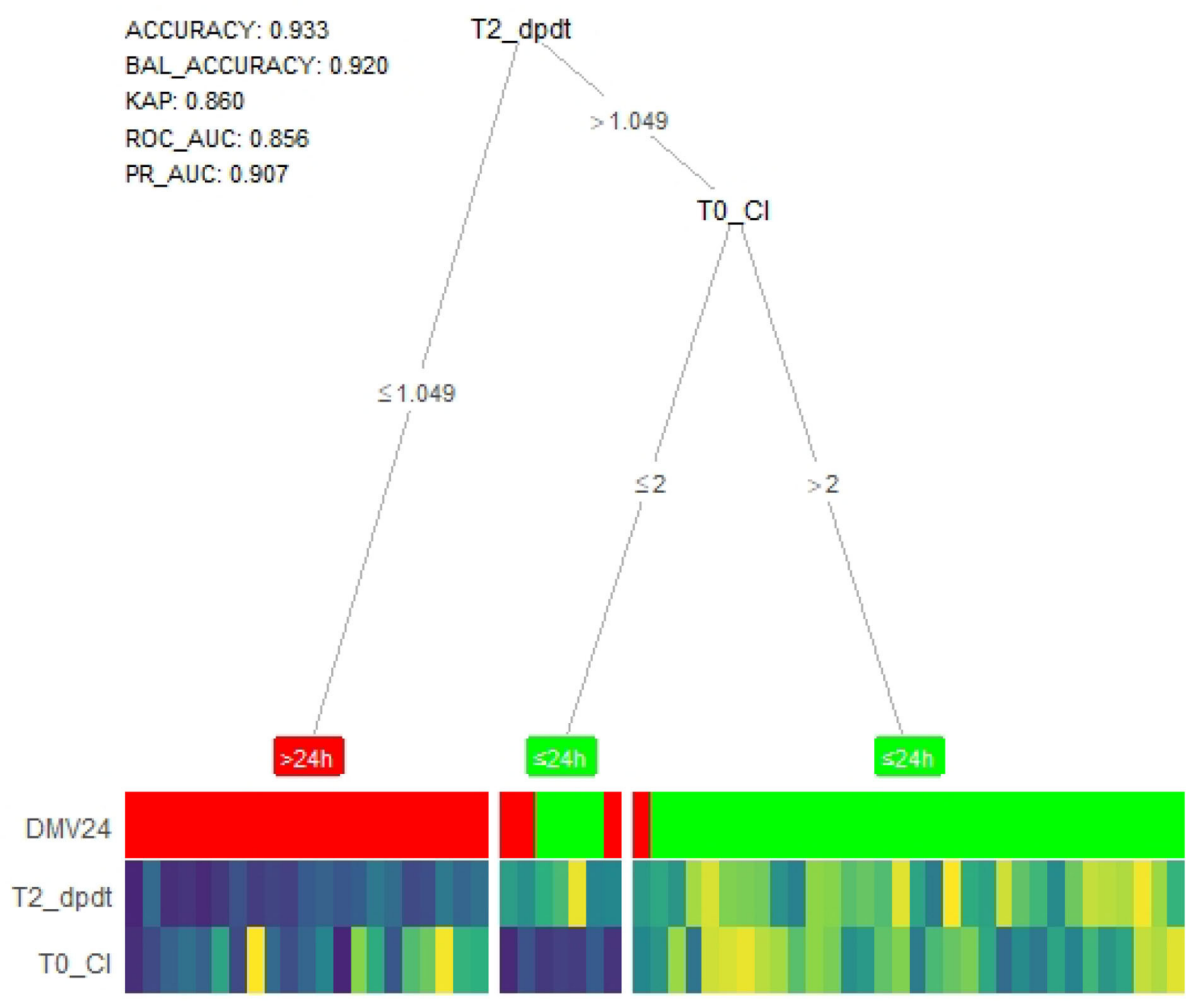
Tree-heat map of XGBoost machine learning model for prediction. T2_dpdt, dp/dt_max_ (the maximal slope of systolic upstroke) at T2. T0_CI, CI (cardiac index) at T0. DMV, duration of mechanical ventilation. BAL_ACCURACY, balance accuracy, KAP, kappa. ROC_AUC, Under area curve of ROC.

The group effect on hemodynamic parameters revealed by the linear mixed model suggested that patients with a prolonged DMV showed significantly lower CI and dp/dt_max_ after the surgery (Table 2, Figure 1). Cardiac dysfunction is the main risk factor for 80% weaning failure (5). Han et al. and Kadir et al. also found that cardiac dysfunction is associated with longer mechanical ventilation duration (30, 31). Both the preload and afterload increase due to the decrease in intrathoracic pressure premature in the process of extubation and increase cardiac work and myocardial oxygen consumption. As a result, premature extubation deteriorates patients' condition by inducing pulmonary edema, pulmonary artery spasm, severe anoxia, heart failure, and reintubation.

dp/dt_max_ measured 8h after the surgery had strongest correlation with a prolonged DMV and was the best hemodynamic predictor (Table 2, Figures 2–4). On the one hand, dp/dt_max_ has generally been used as a sensitive index of cardiac contractility and reserving ability (32). Arterial dp/dt_max_ tracks the left ventricular contractility changes and is mainly determined by myocardial contractility with very limited influence by loading conditions (32–35). Decreased dp/dt_max_ is associated with a myocardial injury even in extracardiac surgery using PRAM (36). Yang et al. also reported that not CI but dp/dt_max_ have a higher correlation with BNP when used PRAM after CHD operation (22). In addition, the exact 8 h after the surgery may be a turning point for restoring cardiac function or prognosis. Yang et al. and Liu et al. observed the nadir of cardiac output, the minimal central venous oxygen saturation, and the peak of BNP and lactate level at 8h after the surgery (22, 37). Su et al. revealed that the increase of troponin-I beyond 8h after CPB was a strong predictor of postoperative hypoperfusion in infants (38).

CI reflects the complex outcomes of the endogenous cardiac, neurohumoral responses, and the exogenous inotropic and vasoactive drugs, dependent more on heart rate, preload, and afterload. Based on the heart–lung interaction theory, an increase in cardiac output is considered a positive response to a volume challenge (39, 40). Although it did not have a linear correlation with delayed extubation, CI measured at T0 is a predictor that could not be neglected (Table 2, Figure 4). Furthermore, the difference of CI and dp/dt_max_ between the two DMV groups at T0 alarmed us that inotropic and vasoactive drugs should be used as early as intraoperatively.

CCE is a unique parameter derived from PRAM, which evaluates the compensating interplay of different cardiovascular system compartments, including left and right ventricular contractility, preload and afterload, heart rate, reflected waves, as well as elasticity of great arteries and the ventricular-arterial coupling (24, 39). CCE with no significant changes over time in our study (Table 2) indicates the constant condition of cardiac energy expenditure for compensation to maintain cardiovascular homeostasis after the surgery (31, 41). However, influenced by many factors, CCE is too sensitive and variable: McBride et al. (42) reported the inability of negative-pressure ventilation to reduce HR in sedated extubated patients which meant that other hemodynamic benefit increases did not translate into the improved CCE.

### XGBoost machine learning-based model

XGBoost machine learning model is a widely used technique for a predictive model for its significant accuracy, which is better than many linear models. It is well designed to prevent overfitting by cross-validation and regularization. Furthermore, the more extended corresponding color column of the outcome means more cases of the event and more substantial predictive value of these branches in our XGBoost-based model. Heat map colors present the relative value compared to the rest of the group on each feature (21). Although CI at T2 had good performance in ROC curves, dp/dt_max_ at the same time point probably substitutes for it completely, and CI at T0 improved the predictive value when added into the algorithm. XGBoost machine learning model has been more prevalent in dealing with clinical problems such as treatment evaluation and disease risk management (43).

### Other factors affecting DMV in infants

Age and weight are important factors for both early extubation in fast-track management and delayed extubation after congenital heart surgery in children (44). Infants' left ventricle has altered relaxation characteristics that progressively change over the first year of life and reach adult level (45). The less proliferation of cardiomyocytes and sarcoplasmic reticulum in the myocardium contributes to the lower cardiac contractility. Besides, infants with lower body weight had a higher frequency of adverse events and longer DMV, ICU stay and hospital stay (46, 47). Cardiac surgery often necessitates CBP, which causes myocardial ischemia–reperfusion and induces oxidative stress and ventricular dysfunction (48). Children with a prolonged DMV undergo longer CBP and ACC during the surgery (Table 1). However, CBP and ACC did not correlate with a prolonged DMV and could not use for prediction (Figures 2, 4). Tabib et al. and Garci'a-Montes et al. also reported a similar irrelevance to the mechanical ventilation condition (44, 49).

## Limitation

There are several limitations to this study. First, the sample size is not big enough, especially for applying the machine learning model. The number of key factors and their priority may change if more patients are included. Extensive multi-center cohort study including complicated cases is needed to confirm our findings. Our general protocol for determining whether patients should be performed extubation may not align with management at other institutions. In addition, MostCare machine records the data at every 30s, but the calculation on postoperative hours is rough and thus the data collected unavoidably have time error.

## Conclusion

In summary, postoperative hemodynamic management with PRAM shed light on the interconnection between cardiac function and mechanical ventilation. CI measured by PRAM immediately after ICU admission and dp/dt_max_ 8 h later are two key factors in predicting a prolonged DMV with the application of the machine learning model.

## Data availability statement

The original contributions presented in the study are included in the article/Supplementary material, further inquiries can be directed to the corresponding author/s.

## Ethics statement

The studies involving human participants were reviewed and approved by Ethical Committee of Capital Institute of Pediatrics. Written informed consent to participate in this study was provided by the participants' legal guardian/next of kin.

## Author contributions

ML and SW contributed equally to this work and shared co-first authorship. They were responsible for conceptualization/design, methodology, data curation, investigation, and formal analysis. HuZ was responsible for supervision/oversight and funding resources. HoZ, YW, and BM was responsible for drafting the initial manuscript and reviewing or editing the manuscript. All authors contributed to the article and approved the submitted version.

## Funding

This research was supported by the Special Fund of the Pediatric Medical Coordinated Development Center of Beijing Hospitals Authority (XTZD20180301).

## Conflict of interest

The authors declare that the research was conducted in the absence of any commercial or financial relationships that could be construed as a potential conflict of interest.

## Publisher's note

All claims expressed in this article are solely those of the authors and do not necessarily represent those of their affiliated organizations, or those of the publisher, the editors and the reviewers. Any product that may be evaluated in this article, or claim that may be made by its manufacturer, is not guaranteed or endorsed by the publisher.
