# Supplementary material for: The predictive value of pressure recording analytical method for the duration of mechanical ventilation in children undergoing cardiac surgery with an XGBoost-based machine learning model
Source: Front Cardiovasc Med. 2022 Oct 28;9:1036340. doi: 10.3389/fcvm.2022.1036340 (PMC9649993; doi:10.3389/fcvm.2022.1036340)
Supplement: Supplementary file 1 [file Table_1.DOCX]

| Supplementary Table 1 Postoperative hemodynamic records of patients with different DMV | | | |
| --- | --- | --- | --- |
|  | | DMV≤ **24**h (n=35) | DMV> **24**h (n=25) |
| CI  (L/min/m^2^) | T0 | 2.55±0.39 | 2.28±0.59 |
|  | T1 | 2.60±0.40 | 2.32±0.40 |
|  | T2 | 2.99±0.44 | 2.51±0.41 |
|  | T3 | 2.91±0.54 | 2.71±0.62 |
| CCE | T0 | -0.327±0.361 | -0.475±0.468 |
|  | T1 | -0.237±0.330 | -0.315±0.278 |
|  | T2 | -0.296±0.330 | -0.316±0.300 |
|  | T3 | -0.299±0.351 | -0.255±0.273 |
| dp/dt _max_ | T0 | 1.113±0.247 | 0.924±0.289 |
|  | T1 | 1.147±0.210 | 0.920±0.265 |
|  | T2 | 1.296±0.115 | 0.954±0.265 |
|  | T3 | 1.210±0.201 | 1.056±0.218 |
| *DMV, Duration of mechanical ventilation. CI, cardiac index. CCE, cardiac cycle efficiency. dp/dtmax, the maximal slope of systolic upstroke.* | | | |
